# Comparative fecal microbiome analysis of the endangered Volcano rabbit (*Romerolagus diazi*) reveals a microbial core in contrasting habitats of Central Mexico

**DOI:** 10.1371/journal.pone.0343260

**Published:** 2026-03-26

**Authors:** Leslie M. Montes-Carreto, Hanya D. Arellano-Hernández, José Antonio Guerrero, Esperanza Martinez-Romero

**Affiliations:** 1 Laboratorio de Ecología Genómica, Centro de Ciencias Genómicas, Universidad Nacional Autónoma de México, Cuernavaca, Morelos, México; 2 Laboratorio de Monitoreo y Conservación de Fauna, Facultad de Ciencias Biológicas, Universidad Autónoma del Estado de Morelos, Cuernavaca, Morelos, México; UNAM: Universidad Nacional Autonoma de Mexico, MEXICO

## Abstract

Herbivores show a larger microbial diversity in their guts than omnivores or carnivores. Bacterial symbionts expand the host digestion capacity by fermenting cellulose and hemicellulose. Comparisons between populations in different distribution areas can reveal how environmental conditions affect microbiota, helping to design conservation strategies. The Volcano rabbit (*Romerolagus diazi*) is the smallest lagomorph in Mexico. It is classified as endangered by Mexican legislation and as critically endangered by the IUCN, in the Red List. Here we extend our previous microbiome study to another region in Mexico from a high site near an active volcano, the Popocatépetl. In both areas, the most abundant bacterial genera included *Acinetobacter*, *Enterobacter*, *Streptomyces*, *Bacteroides*, *Pseudomonas*, *Janthinobacterium*, *Flavobacterium*, and *Duganella*. Among Archaea, *Methanosarcina*, *Halobaculum*, *Thermococcus*, *Halorubrum*, and *Methanobrevibacter* were prevalent. Fungal genera such as *Fusarium*, *Ascochyta*, *Pyricularia*, *Aspergillus*, and *Colletotrichum* were also identified. Potential functions were identified including carbohydrate, amino acid and nucleotide metabolism. The most abundant enzymes were transferases, hydrolases and oxidoreductases. The PERMANOVA test between areas for Bacteria (p = 0.26), Archaea (p = 0.21) and Fungi (p = 0.48) indicated no significant differences in the taxonomic composition or coding sequences (p = 0.5), although there were differences in relative abundances. Additionally, for archaea, genera that had not been reported previously in Volcano rabbit fecal microbiomes such as *Halomicroarcula*, *Halomicrobium*, *Haloplanus*, and *Sulfolobus* were identified, with *Sulfolobus* found exclusive in Izta-Popo. The Volcano rabbit fecal microbiome showed unique bacterial and archaeal profiles. Overall, these microbial communities are likely to contribute to the digestion of plant fibers, phenolic compounds, and other dietary components, underscoring their importance for the health and conservation of these endangered species.

## Introduction

The microbiome encompasses the collective genomes of microorganisms, including fungi, bacteria, and archaea, that inhabit a host, forming dynamic ecosystems of trillions of microbes in different organs and tissues [1–4]. In recent years, there has been an increase in knowledge on how these microbial communities perform essential functions for animal homeostasis, immune system regulation, metabolic, endocrine, and neurological processes [5,6].

The gut microbiome exerts effects that extend beyond the digestive tract through its involvement in the gut–brain axis. This axis includes neurotransmitters, hormones, and microbial metabolites such as short-chain fatty acids (SCFAs), which help regulate cognitive functions, stress responses, and social behavior [7–9]. The microbiome can also influence the synthesis and availability of neurotransmitters like serotonin, dopamine, and GABA, thereby affecting processes such as memory formation and appetite regulation [10].

When the composition of these microbial communities shifts due to internal or environmental factors, an imbalance known as dysbiosis may occur. This condition is characterized by a reduction in microbial diversity, the growth of opportunistic or pathogenic taxa, and the loss of key symbiotic species that produce beneficial metabolites, which are associated with a wide range of inflammatory, metabolic, autoimmune, and neurological diseases [11]. In wild animals, dysbiosis can compromise the host’s ability to adapt to its environment, increase susceptibility to infections, and alter its behavior, which could eventually impact its reproductive success and survival [12]. Therapeutic strategies, such as fecal microbiota transplantation and dietary modulation, have been developed to restore microbial balance and improve host health [11].

The gut microbiome plays an essential role in mammals by contributing to digestion and nutrient absorption [13–15]. Several factors have been identified as key determinants that modulate the composition and function of the mammalian gut microbiome, including host phylogeny [16], physiology [17], diet [18], behavior [19], geographic location and habitat quality [20,21].

Diet plays a central role in shaping the gut microbiome, either by providing nutrients that support microbial growth or by directly introducing microbial taxa [4,18,22]. Compared to other mammals, rabbits exhibit a gastrointestinal (GI) microbiome that is uniquely adapted to their hindgut fermentation physiology and cecotrophy, a specialized behavior in which they re-ingest soft fecal pellets to maximize nutrient absorption [23]. The plant origin of many gut microbes has been observed especially in herbivores such as rabbits [22]. The rabbit gut microbiome includes bacteria that encode tannase enzymes capable of degrading plant polyphenols, as well as methanogenic archaea that possess *nifH* genes involved in nitrogen fixation [24].

The order lagomorpha includes 93 species of pikas, rabbits and hares [25]. The diversity of wild lagomorphs in America comprises at least 17 species adapted to a wide range of ecosystems [26]. Efforts are being made for the conservation of endangered rabbit species. In Mexico, among the most threatened species is the Volcano rabbit (*Romerolagus diazi*). This small lagomorph is endemic to the Transmexican Volcanic Belt, and the only representative of the genus. This species is classified as endangered by Mexican legislation (NOM-059-SEMARNAT) and critically endangered according to the IUCN Red List [27]. It inhabits mountainous areas above 2,800 meters above sea level, particularly in four core areas and 12 peripheral zones located in the Pelado, Tláloc, Popocatépetl and Iztaccíhuatl volcanoes [28]. However, the range of distribution of the Volcano rabbit has decreased drastically due to anthropogenic activities such as habitat loss and fragmentation due to urbanization, land-use change, forest fires, and climate change [29–32]. Studies have shown that individuals in more degraded habitats have higher cortisol levels, suggesting a stress response related to environmental quality [33].

In addition to population monitoring, habitat restoration, and animal genomic data, gut microbiome analysis represents an emerging tool to assess the health status of threatened species. The integration of microbial knowledge into conservation biology allows for a holistic approach that considers not only the organism, but also its microbial communities [12]. Therefore, three years ago we carried out a first study of the fecal microbiome of the Volcano rabbit that inhabits an area in Morelos, Mexico characterized by a landscape of disturbance and fragmented habitat [24,30]. In order to expand this approach and explore how different ecological contexts influence the gut microbiome of the species, in the present study we analyzed the composition of the fecal microbiome of *R. diazi* in two contrasting areas of its distribution: Coajomulco, an area subject to anthropic pressure, and the Iztaccíhuatl-Popocatépetl National Park (Izta-Popo), with a better habitat quality. Our goal is to determine how habitat quality and environmental conditions influence the diversity and composition of the Volcano rabbit gut microbiome, and to discuss the potential of integrating this knowledge into conservation plans focused on habitat health and ecological restoration.

## Methodology

### Study area and sample collection

**Fig 1:**
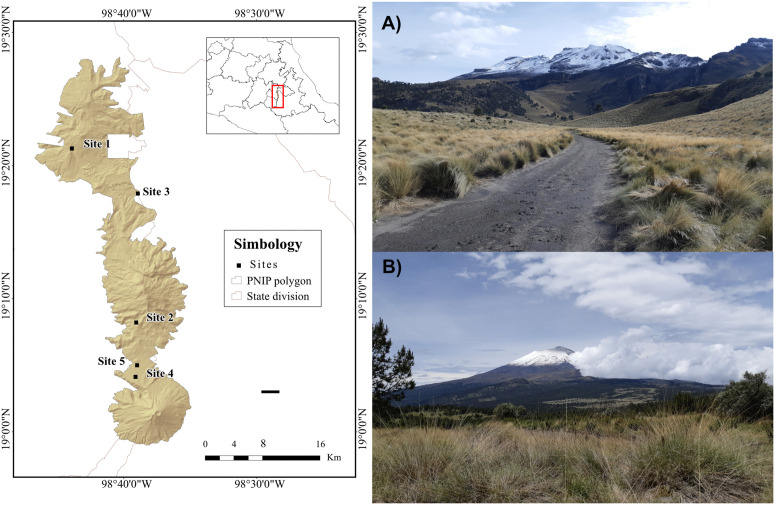
Sampling sites located within the Iztaccíhuatl-Popocatépetl National Park, Mexico: (A) Iztaccíhuatl Volcano; (B) Popocatépetl Volcano. The map was constructed using publicly available spatial layers from INEGI (https://www.inegi.org.mx/app/geo2/elevacionesmex/) and CONABIO (http://geoportal.conabio.gob.mx/metadatos/doc/html/anpjul2025gw.html) and generated in ArcMap v10.8. Photographs of the volcanoes were taken by Leslie M. Montes-Carreto.

Sample collection was conducted early in the day to avoid environmental exposure prior to collection. Eight fresh fecal samples were collected at five sites with Iztaccíhuatl-Popocatépetl National Park, Mexico (14Q UTM 536873.800 m E, 2109107.600 m N) in May 2023 (Fig 1), with permission from Secretaría de Medio Ambiente y Recursos Naturales (SPARN/DGVS/04816/23). The fresh pellets were ochre-colored, with a smooth, glossy texture, firm consistency, a rounded shape with a swollen center, and a maximum diameter of one centimeter.

Natural history observations suggest that Volcano rabbit individuals exhibit limited movement and tend to remain within relatively small home ranges [34]. Within each locality, samples were collected from latrines (defined as accumulations of more than 50 pellets) separated by more than 25 m [34]. This non-invasive method has been used in other population genetics studies of the Volcano rabbit [35,36]. To minimize potential sampling bias, pellets displaying distinct characteristics in size were selected. The fecal samples were placed individually in sterile 1.5 mL Eppendorf tubes, kept on ice in the field, and then placed at 4°C.

### DNA extraction and sequencing

Prior to DNA extraction, fresh fecal pellets were gently rinsed with a stream of sterile distilled water to remove adhering soil particles and other potential surface contaminants. Total DNA from the eight samples was extracted using the Wizard Genomic DNA Purification Kit (Promega, EE. UU). A total of 230 mg of feces was processed per sample. DNA concentration was determined via spectrophotometry using a Nanodrop (Thermo Fisher Scientific, EE. UU) and Qubit™ dsDNA HS Assay Kits (Thermo Fisher Scientific, EE. UU), and quality was assessed in 1.2% agarose gel electrophoresis. The sample requirements for library preparation were as follows: DNA concentration > 200 ng/μL, total DNA amount > 1 μg, and clear, high-quality DNA bands visualized on agarose. Sequencing was performed with Illumina NovaSeq 6000 platform, using paired-end 2 × 150 bp reads, through CD Genomics in New York.

### Bioinformatic analysis

#### Sequence filtering and metagenome assembly.

In addition to sequences from Izta-Popo, sequence data from four previously obtained metagenomic samples of Volcano rabbit from Coajomulco [24] were also analyzed. A total of 710 million raw reads were obtained and after the cleaning process (removing low-quality sequences, short sequences, and adapters), 680.2 million clean reads remained. The sequence cleaning step was performed using the FASTP v0.23.2 program [37] with default parameters plus the following options: --cut_tail, --cut_right, and --cut_mean_quality. Reads were considered high-quality if the Phred quality score was 28 or higher. Clean reads were mapped against genomes from human, rat, mouse, rabbit, pika, nematode, fruit fly, yeast, plants, oak, and adapters to identify any potential contamination using FastQ Screen 0.14.0 [38]. Subsequently, each sample was assembled using SPADES v3.12.0 [39] with the –meta option. Mitochondrial sequences of the Volcano rabbit from Coajomulco metagenomes were previously recovered [40]. To confirm that fecal samples from the Iztaccíhuatl-Popocatépetl National Park area derived from the rabbit species, mitochondrial DNA sequences were identified using Blast 2.10.0+ [41].

### Alpha diversity and microbial composition

Taxonomic assignment per sample was estimated using Kraken2 2.0.8 [42] and eggNOG-mapper v2 [43]. In all subsequent analyses samples were grouped according to two areas with contrasting habitat quality. The Coajomulco area is characterized by fragmented and degraded habitat, where the estimated density of Volcano rabbit was 4.5 individual/ha ranged [44]. In contrast, the Izta-Popo area is dominated by continuous habitat where average annual densities range from 5.6 to 10.8 individuals/ha [45]. Microbial community abundance (Bacteria, Archaea and Fungi) at the genus level was estimated with Bracken 2.5.0 (Bayesian Re-estimation of Abundance with Kraken) with a confidence threshold greater than 98% [46]. Both programs were performed with a Standard Plus Refseq 2024 database containing genomes from bacteria, archaea and fungi. Additionally, a search for eukaryotes using ITS sequences was performed on the Volcano rabbit metagenomes using the UNITE database [47] to recover sequences corresponding to fungi.

Microbial diversity was estimated at the genus level for Bacteria and Archaea using Hill numbers under the same sample coverage [48]. Hill numbers are diversity indices that vary by an exponent q (qD) [49], where q = 0 corresponds to species richness, q = 1 is the exponential of Shannon entropy (effective number of common elements), and q = 2 is the inverse of the Simpson index, representing the effective number of dominant elements [50]. To compare microbial diversity across the contrasting areas, 95% confidence intervals (CIs) were used, where the absence of overlap in CI values indicates significant differences [51]. Diversity qD, sample coverage, and their respective confidence intervals were calculated using the iNEXT package [52] in R 4.4.3 (R Core Team, 2025), with the maximum number of contigs per sample as the endpoint and 1,000 bootstraps for rarefaction curves and CI construction. Additionally, the R package vegan [53] was used to identify core bacteria microbiomes (taxa present in the two contrasting habitats) and exclusive microbiomes (taxa unique to a single habitat), whereas the ggven package [54] was used to create the Venn diagram.

To assess differences in bacterial, archaeal, and fungal community composition between both study areas, a one-way PERMANOVA was performed using Bray–Curtis dissimilarities with 10,000 permutations in PAST 5.2 [55]. Community patterns were further visualized using non-metric multidimensional scaling (NMDS) based on the same Bray–Curtis distance matrix in PAST.

### Prediction of functional annotation

Gene annotation and identification of coding sequences in the fecal metagenomes were performed using PROKKA 1.12 [56]. A cleaning step was carried out on the protein fasta file to remove redundant sequences with CD-HIT. Subsequently, a second annotation of protein sequences was performed using the online tool GhostKOALA 2.2 [57], which provides KEGG Orthology (KO) annotations.

## Results

### Alpha diversity and microbial composition

**Fig 2:**
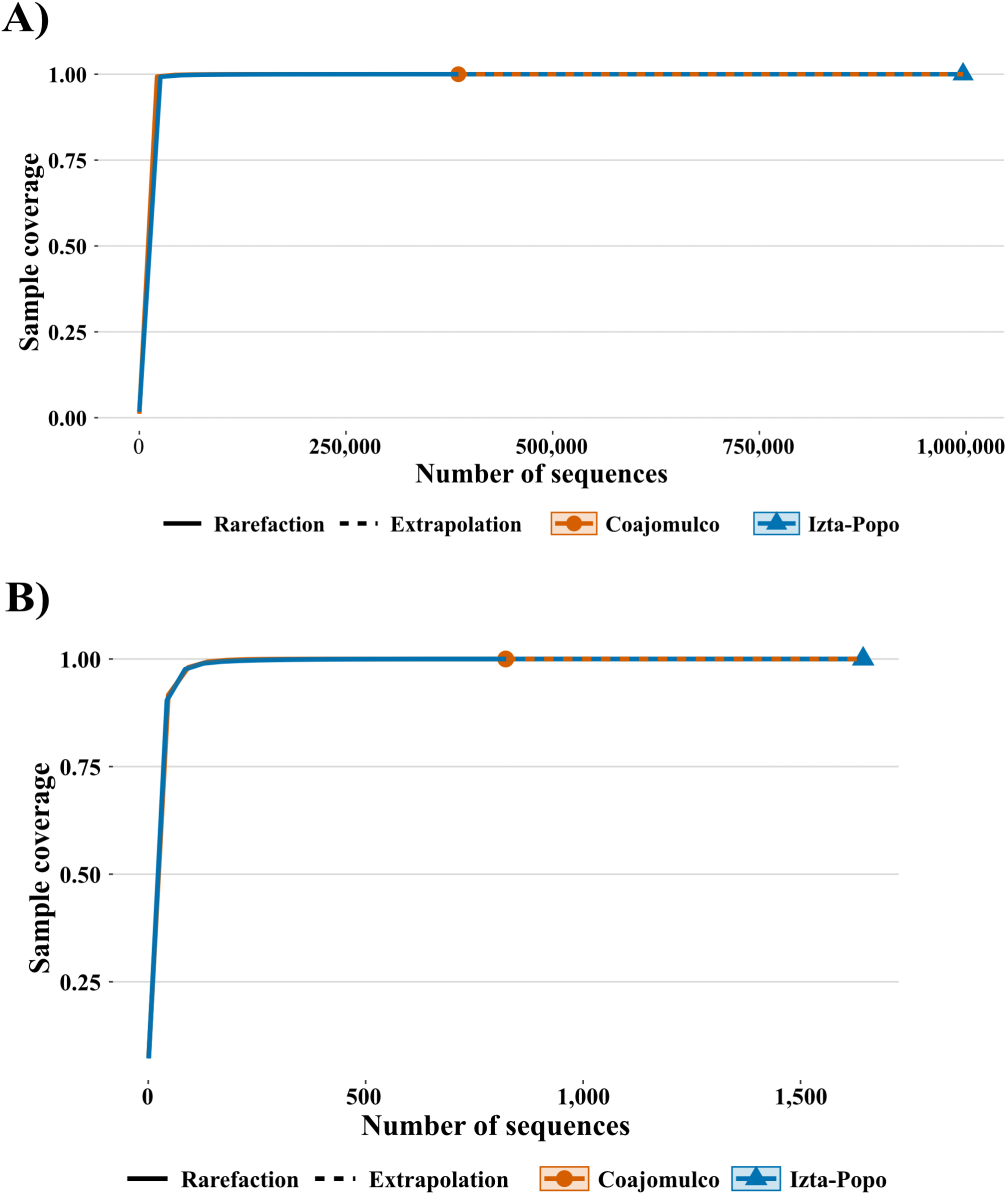
Sampling Coverage (SC) and richness species (q0) for the Bacteria and Archaea domains: (A) Rarefaction curves based on sample coverage from domain Bacteria in both areas (B) Rarefaction curves based on sample coverage from domain Archaea in both areas.

In both areas, at the genus level within the domain Bacteria, sampling coverage was 100%, indicating that sampling was complete. Thus, diversity comparisons were made based on their confidence intervals [48]. Similarly, for all three diversity indices (q = 0, q = 1, and q = 2), coverage was complete in both sites (Figs 2A and 2B).

**Fig 3:**
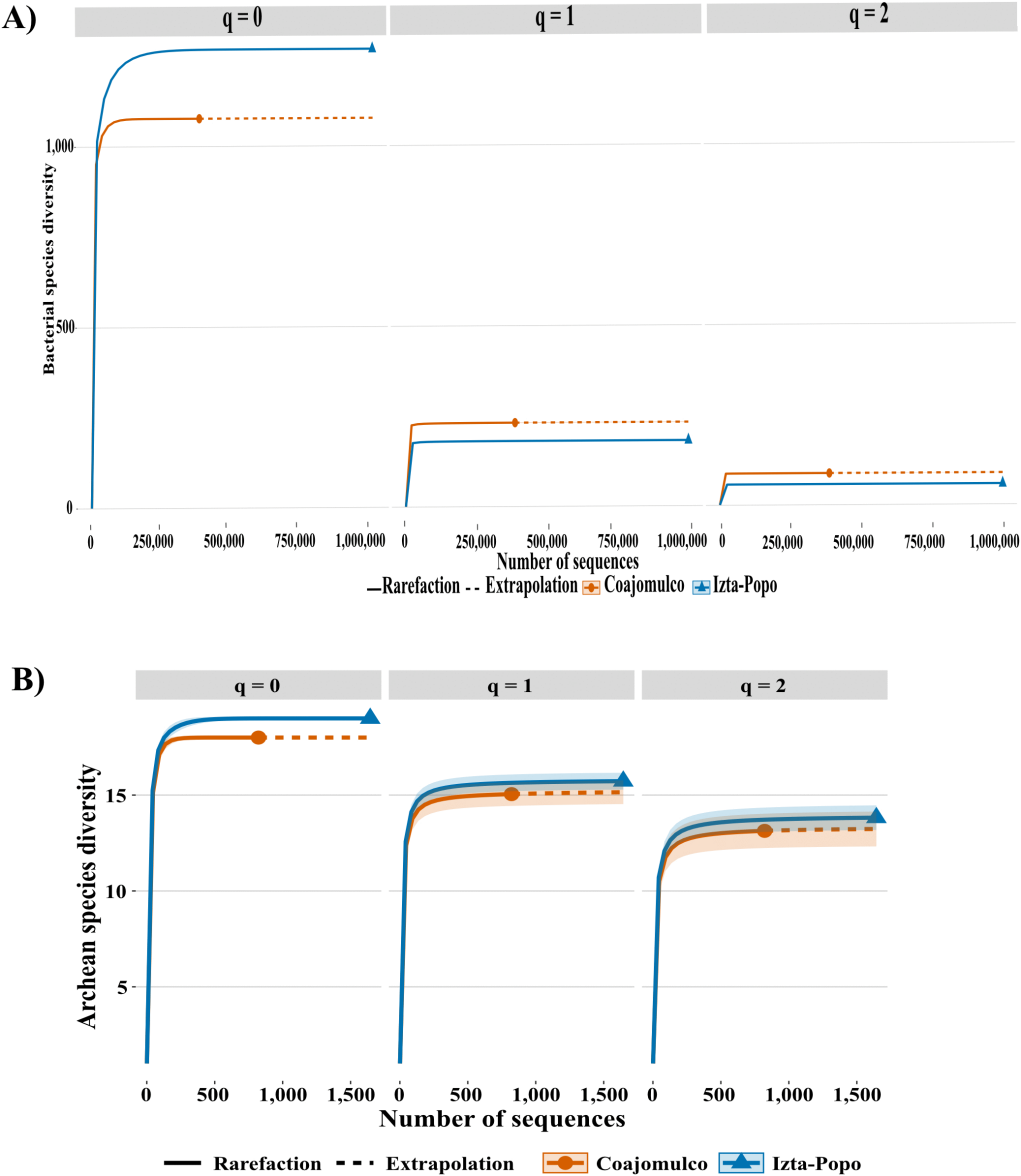
Diversity indices (q0 = richness, q1 = effective number of common genera, q2 = effective number of dominant genera) per area and domain: (A) Rarefaction curves based on the number of sequences per area for the Bacteria domain (B) Rarefaction curves based on the number of sequences per area for the Archaea domain.

At the genus level, bacterial community richness was higher in Izta-Popo (q0 = 1,268) than Coajomulco (q0 = 1,077). However, for equally abundant species (q1) and dominant species (q2), diversity was higher at Coajomulco (q1 = 232, q2 = 88) compared to Izta-Popo (q1 = 181, q2 = 57) (Fig 3A). In contrast, archaeal communities showed slightly higher richness, equally, and dominance at Izta-Popo (q0 = 19, q1 = 16, q2 = 14) than at Coajomulco (q0 = 18, q1 = 15, q2 = 13) (Fig 3B).

**Fig 4:**
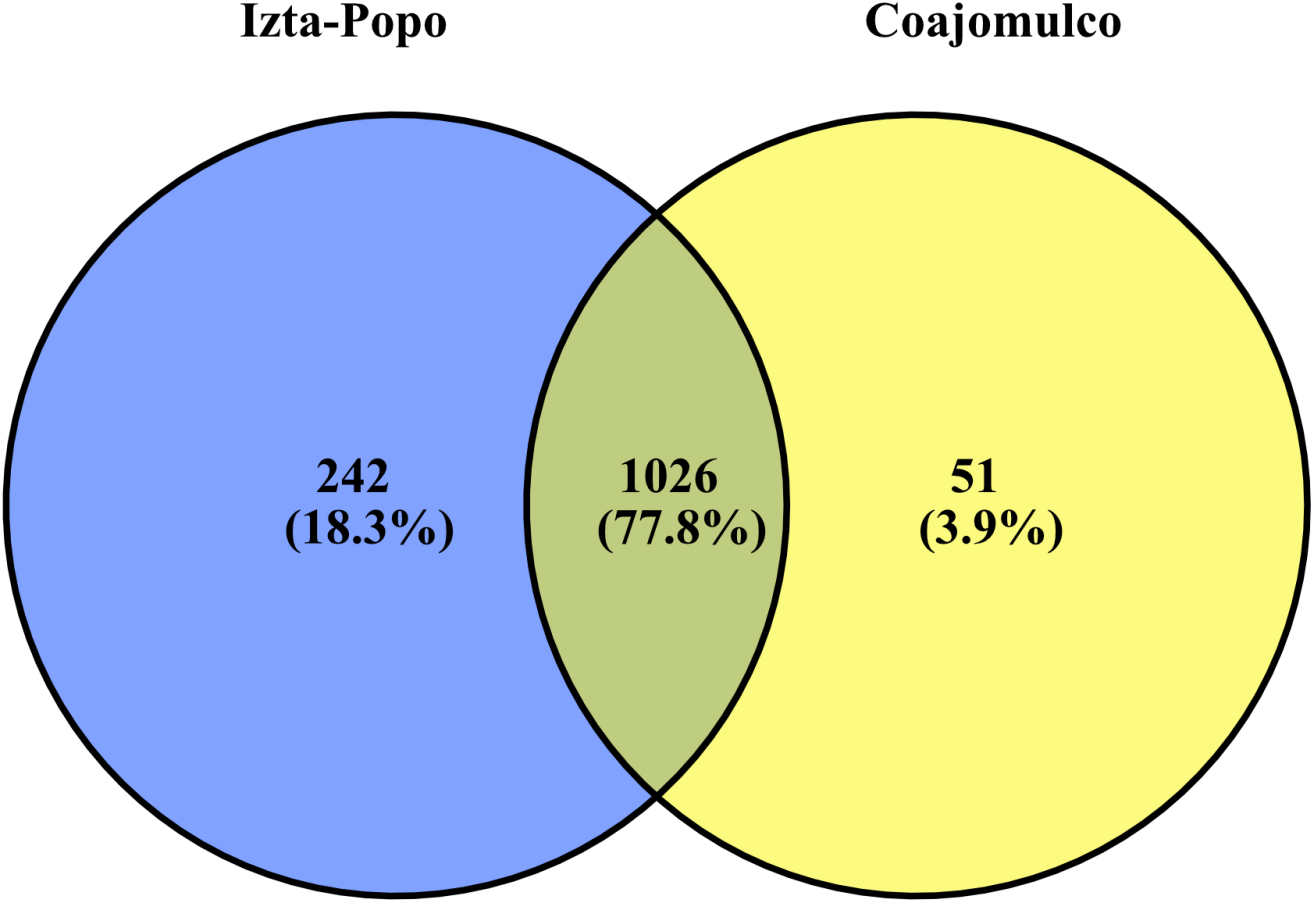
Venn diagram illustrates the core and exclusive bacterial genera found in the Coajomulco and Izta-Popo areas. The shared region indicates genera present at both, while the distinct sections represent area-specific genera.

Analysis of the core community revealed that 1,026 bacterial genera were common in both habitats (Fig 4). The Izta-Popo habitat had 242 unique genera, whereas the Coajomulco habitat had only 51.

**Fig 5:**
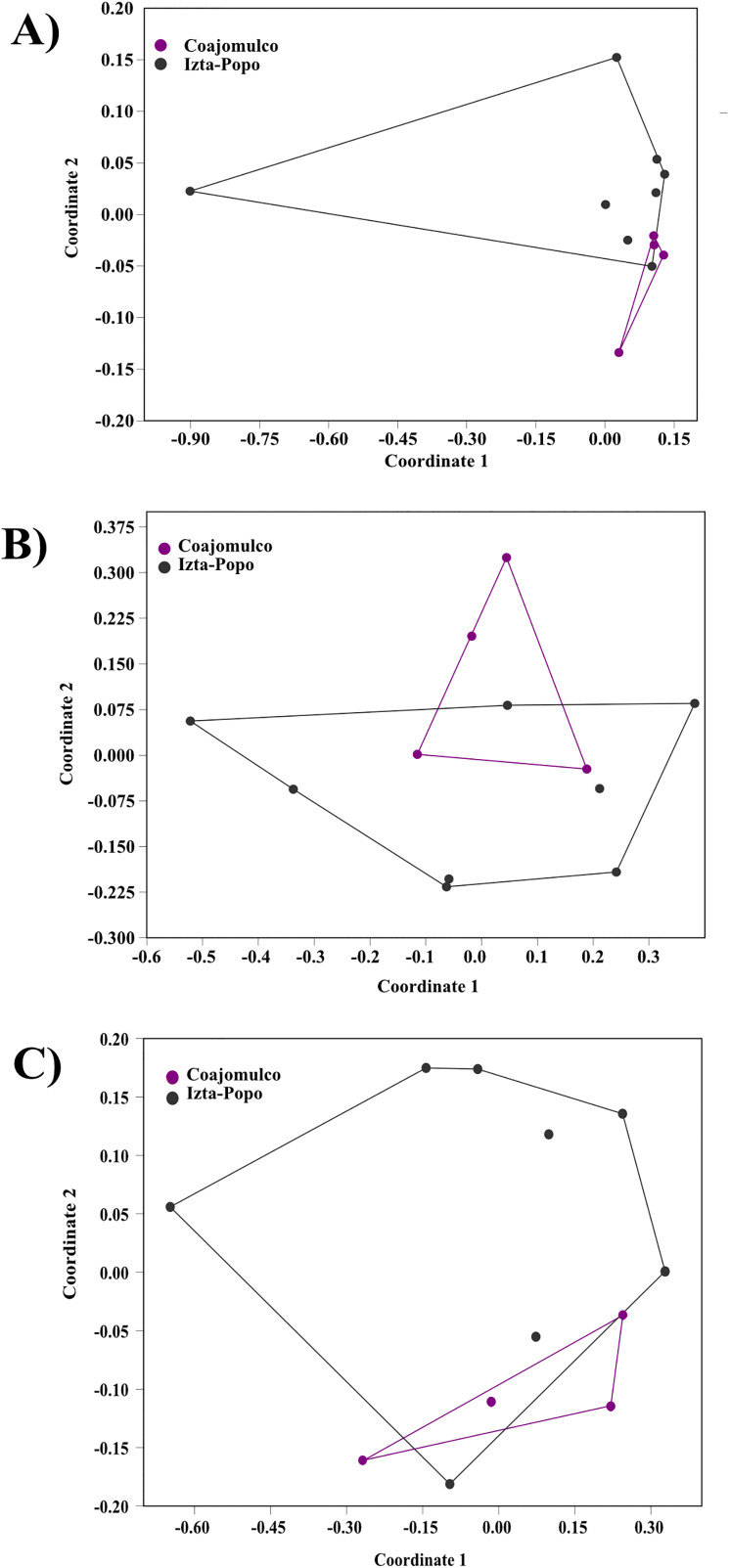
Non-metric Multidimensional Scaling (NMDS) ordination of fecal microbial communities based on Bray–Curtis dissimilarities. Ordinations are shown for (A) bacterial, (B) archaeal, and (C) fungal communities from the two study areas. Each point represents an individual fecal sample.

According to PERMANOVA, no significant differences in microbial community composition at the genus level were detected between contrasting habitat areas for Bacteria (p = 0.26), Archaea (p = 0.21), or Fungi (p = 0.48). Consistently, non-metric multidimensional scaling (NMDS) ordinations based on Bray–Curtis dissimilarities showed overlap among samples from both areas, indicating similar community structures across habitats (Figs 5A–5C).

In metagenomes from the Coajomulco area previously analyzed (Montes-Carreto et al., 2021), a total of 24 bacterial phyla were identified, with Pseudomonadota (Proteobacteria), Bacillota (Firmicutes), Actinomycetota (Actinobacteria), and Cyanobacteriota (Cyanobacteria) being the most prevalent. In comparison, the metagenomes from the Izta-Popo area revealed 19 phyla, mainly Pseudomonadota (Proteobacteria), Bacillota (Firmicutes), Actinomycetota (Actinobacteria), and Bacteroidota (Bacteroidetes).

At both areas, three archaeal phyla were consistently detected: Euryarchaeota, Thermoproteota (Crenarchaeota), and Nitrososphaerota (Thaumarchaeota), with Euryarchaeota being the most abundant, consisting mainly of methanogenic archaea present in both areas.

**Fig 6:**
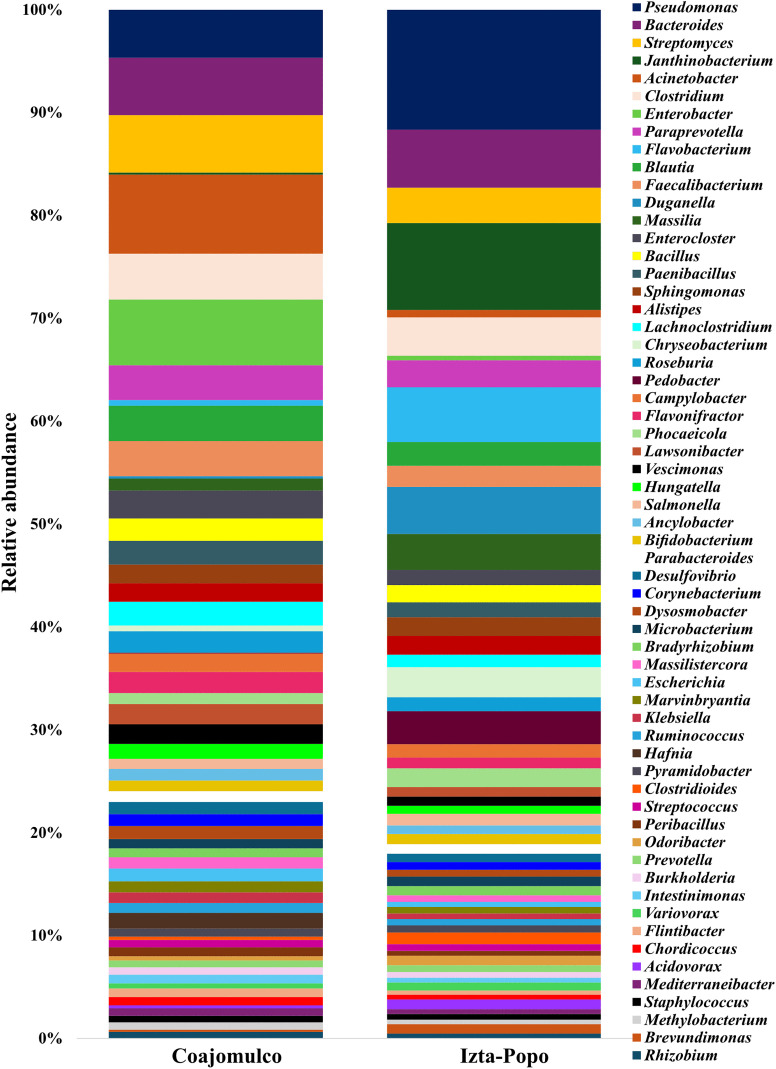
Relative abundance (%) of the 60 most representative bacterial genera identified at the Coajomulco and Iztaccíhuatl-Popocatépetl National Park (Izta-Popo) areas.

Regarding bacterial genera, we identified 1,319 genera. In Coajomulco, the most abundant were *Acinetobacter* (Pseudomonadota) (7%), *Enterobacter* (Pseudomonadota) (5.8%), *Streptomyces* (Actinomycetota) (5.1%), Bacteroides (Bacteroidota) (5%), and *Pseudomonas* (Pseudomonadota) (4.2%). In contrast, in Izta-Popo, the dominant genera were *Pseudomonas* (Pseudomonadota) (10.8%), *Janthinobacterium* (Pseudomonadota) (7.8%), *Bacteroides* (Bacteroidota) (5.2%), *Flavobacterium* (Bacteroidota) (4.9%), and *Duganella* (Pseudomonadota) (4.2%) (Fig 6).

**Fig 7:**
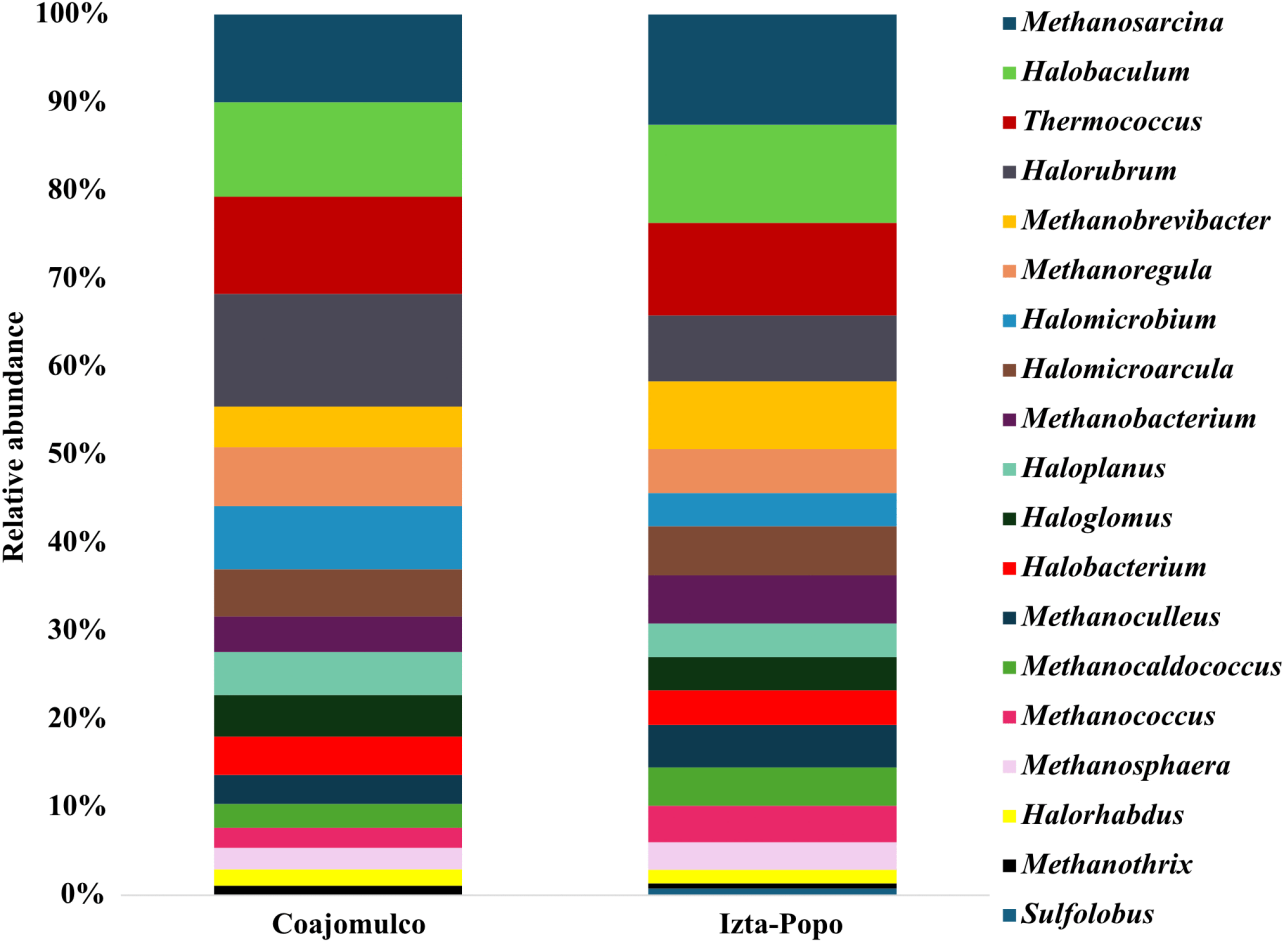
Relative abundances (%) of the archaeal genera identified in samples from the Coajomulco and Izta-Popo areas.

We identified 19 genera within the Archaea domain. Among these, *Methanosarcina* (12.5%−9.9%), *Halobaculum* (11.13%−10.71%), *Thermococcus* (11%−10.5%), *Halorubrum* (12.7%−7.5%), *Methanobrevibacter* (7.6%−4.6%), and *Halomicrobium* (7.1%−3.7%) were the most abundant across both areas. Additionally, other genera such as *Halomicroarcula* (5.6%−5.3%), *Haloplanus* (4.8%−3.8%), and *Sulfolobus* (0.7%), were found with *Sulfolobus* exclusive in Izta-Popo (Fig 7).

A total of 4,631 fungal sequences were obtained from both sampling areas. These sequences were grouped into two main phyla: Ascomycota and Basidiomycota, with the former being the most prevalent in both habitats. In total, 34 fungal genera were identified, among which *Fusarium* (23.32%–18.94%), *Ascochyta* (17.67%–8.75%), *Pyricularia* (10.84%–7.42%), *Aspergillus* (7.56%–9.17%), and *Colletotrichum* (9.47%–7.08%) showed the highest relative abundances (S1 Fig). It is worth noting that while all 34 genera were detected at Izta-Popo, only 18 genera were identified at Coajomulco, indicating lower fungal diversity at this area. Additionally, several genera were exclusively found at Izta-Popo, including *Eremothecium* (6.43%), *Saccharomyces* (3.34%), and *Zymoseptoria* (1.81%), as well as others at lower abundance, such as *Naumovozyma*, *Purpureocillium*, *Psilocybe*, *Trichoderma*, *Cryptococcus*, and *Malassezia* (S1 Fig).

### Functional annotation prediction

**Fig 8:**
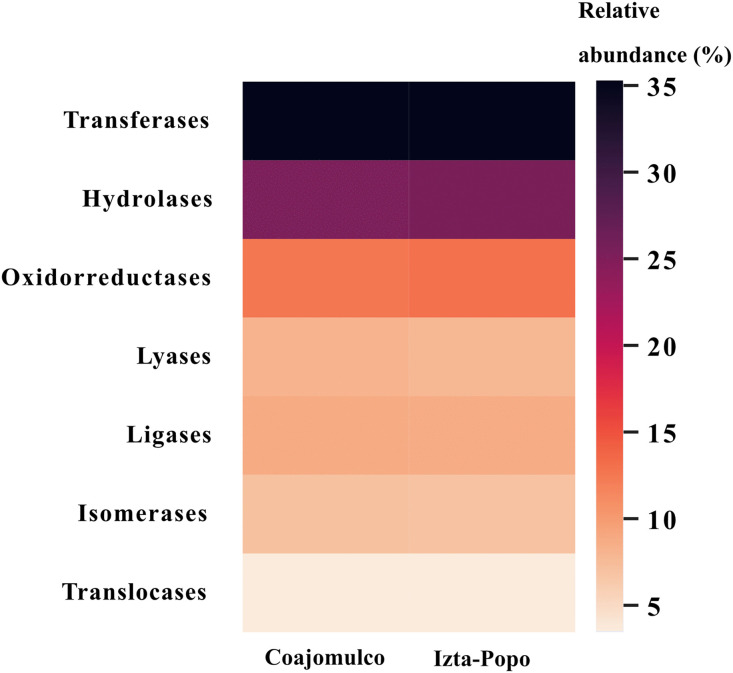
Relative abundance (%) from enzymes found in Volcano rabbit metagenomes from the Coajomulco and Izta-Popo areas.

The metagenomes primarily revealed coding regions (CDS) (98%), rRNA (0.1%), tRNA (1.5%), and tmRNA (0.02%) in both areas S1 Table. Additionally, sequences were identified for enzymes classified according to their specific catalytic functions (Schomburg et al., 2017). In both zones the most abundant genes were those encoding transferases (35% relative abundance) and hydrolases (25%), followed by oxidoreductases (12.5% − 13%), lyases (8% − 7.7%), ligases (8.7% − 8.6%), isomerases (6.9%), and translocases (3.4%) (Fig 8).

**Fig 9:**
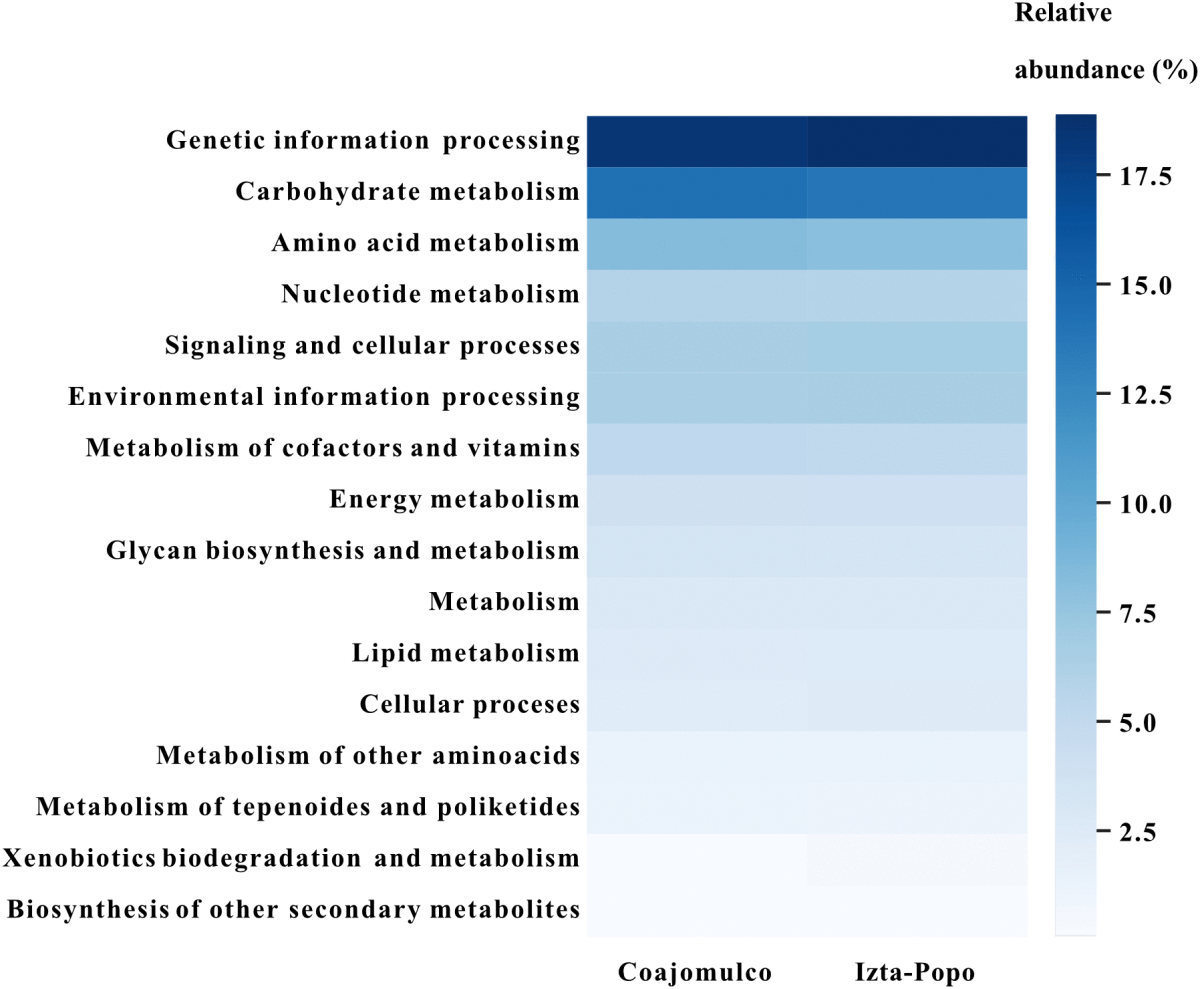
Comparison of relative abundance (%) of functional categories across different Volcano rabbit areas.

Finally, the metagenomes were found to encompass 16 functional categories reported in the KEGG database. The most abundant categories across samples were genetic information processing (18% relative abundance), carbohydrate metabolism (glycolysis/gluconeogenesis, citric acid cycle, TCA cycle 14%), amino acid metabolism (serine and threonine, aromatic amino acid, cysteine and methionine metabolism and others; 8%) and nucleotide metabolism (purine and pyrimidine metabolism; 5.8%). In contrast, xenobiotic biodegradation (aromatics degradation; 0.8% − 0.03%), and biosynthesis of other secondary metabolites (beta-lactams, other bacterial compounds; 0.1%) had the fewest annotated sequences (Fig 9).

## Discussion

This study offers a comprehensive metagenomic overview of the bacterial, archaeal and fungal communities associated with the habitat of the endangered Volcano rabbit (*Romerolagus diazi*) in two areas with contrasting habitat quality [44,45]. Overall, the complete sampling coverage achieved for both Bacteria and Archaea supports the reliability of the diversity estimates and indicates that the sequencing effort was sufficient to obtain the majority of microbial taxa present [58].

Regarding bacterial communities, the core microbiome observed in both areas was similar despite differences in vegetation, soil properties, or human activity [59]. While habitat disturbance is often linked to reduced gut microbial diversity in many wildlife species [60], the Volcano rabbit’s gut microbiome may reflect unexpected patterns of diversity in relation to habitat with greater anthropic pressure like Coajomulco. Evidence shows that changes in land use and food resources can increase microbial richness like in the rocky mountain snail (*Oreohelix strigosa*) [20], the yellow baboon (*Papio cynocephalus*) [61], olive baboons (*Papio anubis*) [62], due to a more varied diet including new plant species introduced by anthropogenic activities like agriculture or human-derived diet. This is supported by the fact that the Volcano rabbit diet in Coajomulco includes 37 plant species [63] while in Izta-Popo there are only 15 species [64]. Genera such as *Acinetobacter*, *Enterobacter*, *Streptomyces*, and *Pseudomonas* are known for their metabolic versatility, roles in nutrient cycling, and capacity to interact with plants and animals [65].

In contrast, archaeal diversity showed a slight increase in richness and dominance at the Izta-Popo. The abundance of methanogenic and thermophilic archaea across the two different areas highlights their stable presence, which could play essential roles in soil carbon cycling and nitrogen transformations [66]. Archaea could represent the stable microbiota in these rabbits and could have a maternal origin. On the other hand, the genus *Sulfolobus* was found at Izta-Popo, possibly reflecting localized environmental conditions, such as higher soil acidity or geothermal influence, that support its growth [67]. The dominant genera like *Methanosarcina* and *Methanobrevibacter* are known to participate in methanogenesis pathways, which may be significant for carbon fluxes in mountain soils [68]. These genera use one- or two-carbon compounds such as CO₂, H₂, and formate (HCOO), methanol, or acetate (CH_3_COO-), which are derived from bacterial metabolism and the degradation of fiber-rich plant matter [69,70].

Fungal communities “Micobiota” were dominated by Ascomycota, with *Fusarium*, *Ascochyta*, and *Aspergillus* genera previously reported as endophytes of plants [71,72]. Fungi have been reported in diets high in vegetable fiber [73,74]. Studies on animals have shown that they naturally include fungi as part of their diet [75,76]. Because these fungi are ubiquitous, it is expected that they will be found on and inside hosts and may become part of the gut microbiome, although in lower abundance than bacteria. Previous studies have mainly reported the phyla Ascomycota and Basidiomycota in the human and bat gut microbiome [74,77,78], while in other mammals such as mice and primates, Chytridiomycota and Zygomycota have also been reported [61,79]. Moreover, fungal richness was higher at Izta-Popo than Coajomulco, where several genera were exclusively detected in Izta-Popo (*Eremothecium*, *Saccharomyces*, *Zymoseptoria, Psilocybe*, among others). This finding may reflect differences in local vegetation, moisture, or microhabitat complexity that favor the establishment of a wider range of fungi [80]. This finding was reported to red colobus (*Piliocolobus gordonorum*) where fungi were enriched in protected forest [61]. Grieneisen proposed that soil characteristics can shape the microbial communities of both the soil itself and the terrestrial animals inhabiting that environment [81]. It has been reported in metagenomic studies from high-altitude soils that environmental heterogeneity promotes diverse endophytic and saprophytic assemblages of fungi [82].

At the functional level, from Bacteria and Archaea, the abundance of genes coding for transferases, hydrolases, and oxidoreductases indicates that key metabolic pathways related to organic matter degradation, nutrient cycling, and energy transfer are well represented in both areas. The predominance of pathways related to carbohydrate, amino acid and nucleotide metabolism aligns with the metabolic versatility expected in soils rich in organic input from plants. These findings are consistent with other hindgut fermenting herbivores like african elephant (*Loxodonta africana*), capybara (*Hydrochoerus hydrochaeris*), european rabbit (*Oryctolagus cuniculus*), among others where carbohydrate, amino acid and nucleotide metabolism had been reported [4,83].

Together, these findings show how local environmental conditions shape the structure and functions of microbial communities. Although both areas share core taxa, unique genera and functional profiles emphasize the need for continued monitoring to understand how these microbial communities contribute to ecosystem resilience and the conservation of endemic species such as the endangered Volcano rabbit.

## Conclusions

This study provides one of the first comprehensive characterizations of the bacterial, archaeal and fungal communities associated with the habitat and fecal microbiome of the endangered Volcano rabbit. The high diversity observed across the zones studied highlights the importance of local environmental factors, such as vegetation, soil properties, and human influence, in shaping microbiome composition and function. We found a consistent presence of key bacterial and archaeal genera involved in nutrient cycling and methanogenesis.

These findings emphasize the need to preserve high-quality, undisturbed habitats to maintain healthy and resilient microbial communities that can directly affect the fitness and survival of this endemic lagomorph. Microbiome monitoring may serve as an early-warning tool to detect habitat degradation and support evidence-based management actions to safeguard the Volcano rabbit and its habitat.

## Supporting information

S1 Table Comparison of relative abundance (%) of functional categories (per area and percentage) identified in Volcano rabbit metagenomes. (DOCX)

S1 Fig Relative abundance (%) of fungal genera identified in metagenomic fecal samples from *Romerolagus diazi* from Coajomulco and Izta-Popo areas. (TIF)
