# Supplementary material for: Comparative fecal microbiome analysis of the endangered Volcano rabbit (Romerolagus diazi) reveals a microbial core in contrasting habitats of Central Mexico
Source: PLoS One. 2026 Mar 26;21(3):e0343260. doi: 10.1371/journal.pone.0343260 (PMC13020824; doi:10.1371/journal.pone.0343260)
Supplement: S1 Table — (DOCX) [file pone.0343260.s001.docx]

| **Categories** | **Coajomulco** | **Izta-Popo** |
| --- | --- | --- |
| **CDS** | 98.34 | 98.31 |
| **rRNA** | 0.13 | 0.12 |
| **tmRNA** | 0.02 | 0.02 |
| **tRNA** | 1.52 | 1.55 |
